# A Reinforcement Ensemble Learning Method for Rolling Bearing Fault Diagnosis under Variable Work Conditions

**DOI:** 10.3390/s24113323

**Published:** 2024-05-23

**Authors:** Yanning Li, Yi Zhang, Ruixin Wang, Jiangfeng Fu

**Affiliations:** 1 School of Automation, Northwest Polytechnic University, Xi’an 710072, China; lynx3333@163.com (Y.L.); zzm1026@126.com (Y.Z.); 2 Xi’an Modern Control Technology Research Institute, Xi’an 710065, China; 3 Key Laboratory of Road Construction Technology & Equipment Ministry of Education, Chang’an University, Xi’an 710061, China; 4 Advanced Power Research Institute, Northwest Polytechnic University, Xi’an 710072, China; fjf@nwpu.edu.cn

**Keywords:** rolling bearing, fault diagnosis, reinforcement learning, ensemble learning

## Abstract

Ensuring the smooth operation of rolling bearings requires a precise fault diagnosis. Particularly, identifying fault types under varying working conditions holds significant importance in practical engineering. Thus, we propose a reinforcement ensemble method for diagnosing rolling bearing faults under varying working conditions. Firstly, a reinforcement model was designed to select the optimal base learner. Stratified random sampling was used to extract four datasets from raw training data. The reinforcement model was trained by these four datasets, respectively, and we obtained four optimal base learners. Then, a sparse ANN was designed as the ensemble model and the reinforcement learning model that can successfully identify the fault type under variable work conditions was constructed. Extensive experiments were conducted, and the results demonstrate the superiority of the proposed method over other intelligent approaches, with significant practical engineering benefits.

## 1. Introduction

Modern large rotating machinery often runs in extreme environments, and in the case a failure occurs, the consequences will be catastrophic. And because rolling bearings are an important part of rotating machinery, it is vital to ensure their safe operation [1]. Hence, the precise identification of the health status of rolling bearings is crucial for the smooth and effective operation of large machinery and equipment [2]. Particularly in the face of the current increasingly complex working conditions, rolling bearings often need to operate at different speeds and under different loads, and so it has become a new challenge to accurately identify the operating conditions under different work conditions.

Currently, transfer learning is often used to conduct fault diagnosis problems under different work conditions. For instance, Wu et al. introduced a deep transfer maximum classifier discrepancy approach for diagnosing rolling bearing faults [3]. He et al. proposed a transfer fault diagnosis method using an enhanced deep auto-encoder [4]. However, these methods still rely on data distribution and cannot intelligently extract similar fault features through the network structure.

Recently, deep learning models have replaced shallow network models and have been extensively applied in fault diagnoses with remarkable results [5,6,7,8]. For instance, Lin et al. proposed a counter-based open-circuit switch method for a single-phase cascaded h-bridge multilevel converter [9]. Zhao et al. proposed a life prediction method based on a digital twin-driven model [10]. Mochammad et al. designed a bearing fault degradation model based on a multitime window fusion unsupervised health indicator [11]. Zhang et al. proposed a model-based analysis method for bearing daults [12].

As the research of intelligent diagnosis methods deepens, the network structure is becoming more and more complex, and selecting the appropriate network model often requires a lot of labor and time costs. Therefore, researchers began to explore automatic methods for building deep learning models based on different tasks [13,14,15]. Currently, reinforcement learning is widely used in this approach due to its powerful autonomous decision-making capabilities. For instance, the Google DeepMind team employed reinforcement learning to discover the optimal neural network structure [16]. Wang et al., on the other hand, introduced a reinforcement-based neural architecture search approach for diagnosing rolling bearing faults [17].

Ensemble learning is an effective deep learning method, and the mainstream ensemble learning methods still combine some weak models to improve the overall performance, and its integration effect mainly depends on the diversity of the models and integration strategies. The ensemble policies are mostly voting methods. For example, Li et al. introduced an improved selective ensemble deep learning approach for fault diagnosis [18]. Feng et al. proposed an ensemble learning method for classifying failure modes and predicting bearing capacity [19]. In this paper, the ensemble learning method is combined with intelligent modules. The first module is a reinforcement learning module, which is used to intelligently select the base model that can identify the same fault type under different operating states. The second module is a sparsely connected artificial neural network (SCANN) model for constructing the ensemble strategies.

Therefore, a Reinforcement Ensemble Learning Method (RELM) is proposed for rolling bearing fault diagnoses under variable work conditions. Firstly, data of identical fault types under varying working conditions are consistently labeled, and four training sets are randomly selected using a reinforcement learning model hierarchically. Secondly, the reinforcement learning model is trained by a policy gradient method, and four different optimal network structures are selected for the four different datasets, and the selected optimal network structures are used as the base models. Finally, a sparse artificial neural network (SCANN) is constructed as an ensemble model, and the output of the base model considered is the input feature of the ensemble model. The proposed method is validated with a high-speed aeronautical bearing dataset; the experimental results demonstrate the effectiveness of the proposed method, and the main contributions are listed as follows.

(1) The RELM is proposed for rolling bearing fault diagnoses under variable working conditions. In contrast to traditional transfer learning methods, the RELM does not need to deal with the data distribution under different work conditions but directly seeks the optimal network structure that can extract key features reflecting the fault condition.
(2) A reinforcement learning method is applied to design an optimal network structure selection model. Intelligent methods are used to replace the significant labor and time costs previously required to design deep learning models.
(3) A SCANN was constructed to effectively reduce the overfitting problem of the secondary classifier.

The rest of this paper is organized as follows: the theoretical basis is introduced in Section 2. The proposed method is explained in detail in Section 3. Experimental verification is carried out in Section 4, and the conclusion is summarized in Section 5.

## 2. Reinforcement Learning

**Figure 1:**
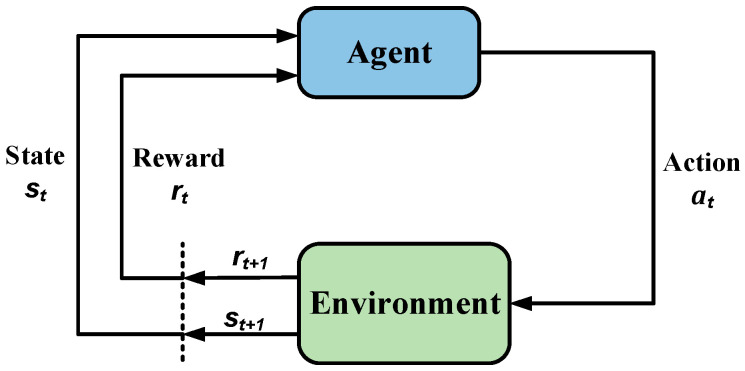
Reinforcement learning.

Reinforcement learning is utilized to describe and solve the task of an intelligent agent learning a policy to reach reward maximization or achieve a specific goal during its interaction with the environment. As depicted in Figure 1, the agent chooses actions, and the environment responds with feedback, causing the agent to transition to a new environment. Simultaneously, the environment provides rewards, and the agent strives to maximize these rewards over time [20].

Reinforcement learning can be viewed as a Markov Decision Process (MDP), representing the interaction between an agent and its environment. This MDP comprises a quadruplet. $MDP=\left(S,A,P,R\right)$: When the agent is in an environment E, S represents the state space. Each state, denoted as s∈S, describes the environment perceivable by the agent, and the agent’s possible actions make up the action space A; when the agent implements action a∈A on the current state s, the environment will transfer from the current state to another state with certain probability P; meanwhile, the environment will give the agent a reward according to the potential reward function R [21].

Hence, a value function is defined to represent the long-term rewards under policy π in the current state. The value function combines the current reward with the cumulative subsequent reward, essentially capturing the expectation of cumulative rewards. The state value function of state s under policy π is denoted as $V^{\pi }(s)$.

(1) $$\begin{array}{c} V^{\pi }\left(s\right)=E_{\pi }\left[\sum_{t=0}^{\infty }\gamma^{t}r_{t}\left|s_{t}=s\right.\right] \end{array}$$

where $E_{\pi }$ represents the expectation under policy π, which is the expected value of selecting actions according to policy π. $\sum_{t=0}^{\infty }\gamma^{t}r_{t}\left|s_{t}\right.$ represents the discounted sum of rewards $r_{t}$ obtained at each time step t. $\gamma \in \left[0,1\right]$ is the discount factor.

Expanding $V^{\pi }\left(s\right)$, in which $r_{t}$ denotes the reward at the future time t and $s^{\prime }$ refers to the next state.

(2) $$\begin{array}{c} V^{\pi }\left(s\right)=E_{\pi }\left[r\left(\left.s^{\prime }\right|s,a\right)+\gamma V^{\pi }\left(s^{\prime }\right)\left|s_{t}=s\right.\right] \end{array}$$

The state $s_{t}$ at moment t and $s_{t+1}$ at moment t+1 are related through a recurrence relation, leading to the deduction of the Bellman equation as follows:

(3) $$\begin{array}{c} V^{\pi }\left(s\right)=E_{\pi }\left[r_{t+1}+\gamma V^{\pi }\left(s_{t+1}\right)\left|s_{t}=s\right.\right] \end{array}$$

Given a policy π and an initial state s, taking action a=π(s) leads to transitioning to the next state $s^{\prime }$, with a probability $P\left(\left.s^{\prime }\right|s,a\right)$. Consequently, the expectation $E_{\pi }$ of the equation can be expanded as

(4) $$\begin{array}{c} V^{\pi }\left(s\right)=\sum_{s^{,}\in S}P\left(\left.s^{,}\right|s,a\right)\left[r\left(\left.s^{,}\right|s,a\right)+\gamma V^{\pi }\left(s^{,}\right)\right] \end{array}$$

where r represents the immediate reward obtained when transitioning from state *s* to state *s*′ by taking action *a*. The initial action, denoted as a, is determined by the policy π and the state s. $G_{t}$ represents the return, which is the cumulative discounted reward obtained after time step t.Taking into account the value influence of action a, the action value function can be expressed as follows:

(5) $$\begin{array}{c} Q^{\pi }\left(s,a\right)=E_{\pi }\left[G_{t}\left|s_{t}=s,a_{t}=a\right.\right]=E\left[\sum_{t=0}^{\infty }\gamma^{t}r_{t}\left|s_{t}=s,\right.a_{t}=a\right] \end{array}$$

Similarly, the Bellman equation for the action value function can be expressed as follows:

(6) $$\begin{array}{c} Q^{\pi }\left(s,a\right)=E_{\pi }\left[r_{t+1}+\gamma Q^{\pi }\left(s_{t+1},a_{t+1}\right)\left|s_{t}=s,a_{t}=a\right.\right] \end{array}$$

The system will transition to the next state $s^{,}$ based on policy π, with a probability denoted as $P\left(\left.s^{\prime }\right|s,a\right)$:

(7) $$\begin{array}{c} V^{\pi }\left(s\right)=\sum_{s^{\prime }\in S}P\left(\left.s^{\prime }\right|s,a\right)\left[r\left(\left.s^{\prime }\right|s,a\right)+\gamma V^{\pi }\left(s^{\prime }\right)\right] \end{array}$$

The optimal policy is determined by maximizing the value function under various initial conditions. This involves finding the policy π that achieves this optimization.

(8) $$\begin{array}{c} V^{*}\left(s\right)=\underset{\pi }{\max}V^{\pi }\left(s\right)=\underset{a}{\max}Q^{*}\left(s,a\right) \end{array}$$

(9) $$\begin{array}{c} Q^{*}\left(s,a\right)=\underset{\pi }{\max}Q^{\pi }\left(s\right)=r+\gamma \sum_{s^{\prime }\in S}P_{ss^{\prime }}V^{*}\left(s^{\prime }\right) \end{array}$$

(10) $$\begin{array}{c} Q^{*}\left(s,a\right)=r+\gamma \sum_{s^{\prime }\in S}P_{ss^{\prime }}\underset{a}{\max}Q^{*}\left(s^{\prime },a\right) \end{array}$$

In which *Pss*′ represents the transition probability from state *ss* to state *s*′ under the action a. In order to fit $V^{\pi }\left(s\right)$ and $Q^{\pi }\left(s,a\right)$ by the parameter θ. A function of the neural network is used to approximate $V_{\pi }\left(s\right)$ and $Q^{\pi }\left(s,a\right)$ [22].

(11) $$\begin{array}{c} V_{\theta }\left(s\right)\approx V^{\pi }\left(s\right) \end{array}$$

(12) $$\begin{array}{c} Q_{\theta }\left(s,a\right)\approx Q^{\pi }\left(s,a\right) \end{array}$$

Parameterize the policy as $\pi_{\theta }\left(s,a\right)=Ρ\left(\left.a\right|s,\theta \right)$, use the model-free approach, put the agent into an uncertain dynamic environment, and use the initial value method for the objective function:

(13) $$\begin{array}{c} J\left(\theta \right)=V^{\pi \theta }\left(s\right)=E\left(r_{t+1}+\gamma r_{t+2}+\gamma^{2}r_{t+3}+\cdots |\pi_{\theta }\right) \end{array}$$

In which $J\left(\theta \right)$ represents the expectation operator under the policy *πθ*. To maximize $J\left(\theta \right)$, that means, seek a set of parameter vectors θ to maximizes $J\left(\theta \right)$. Gradient descent is used to calculate $\nabla_{\theta }J\left(\theta \right)$:

(14) $$\begin{array}{c} \nabla_{\theta }J\left(\theta \right)=\left(\begin{array}{c} \frac{\partial J\left(\theta \right)}{\partial \theta_{1}}\\ \vdots \\ \frac{\partial J\left(\theta \right)}{\partial \theta_{n}} \end{array}\right) \end{array}$$

For multi-step MDPs, the policy gradient method is approached by the likelihood rate, where the value function is the sum of the multi-step values, using $Q_{\pi }\left(s,a\right)$ to replace the reward value $R\;\left(s,a\right)$ of a single step:

(15) $$\begin{array}{c} \nabla_{\theta }J\left(\theta \right)=E_{\pi \theta }\left[\nabla_{\theta }log\pi_{\theta }\left(s,a\right)Q^{\pi \theta }\left(s,a\right)\right] \end{array}$$

The Monte Carlo policy gradient method using stochastic gradient ascent method to update the parameter θ by sampling; the updated formula:

(16) $$\begin{array}{c} \theta_{t+1}\leftarrow \theta +\alpha \nabla_{\theta }log\pi_{\theta }\left(s_{t},a_{t}\right)v_{t} \end{array}$$

Add a baseline function $b\left(s\right)$, the equation can be modified to

(17) $$\begin{array}{c} \nabla_{\theta }J\left(\theta \right)=E_{\pi \theta }\left[\nabla_{\theta }log\pi_{\theta }\left(s,a\right)\left[Q^{\pi \theta }\left(s,a\right)-b\left(s\right)\right]\right] \end{array}$$

## 3. The Proposed Method

### 3.1. The Generation of Baseline Model

At present, deep learning methods are widely used and have achieved good results in fault diagnosis. However, how to select the optimal hyperparameters of a neural network is still a difficult task. Some optimization methods have been used to optimize the network structure but they have not been widely used due to efficiency and network structure constraints [23,24,25]. However, due to the variable data distribution of different datasets and the black box characteristics of the deep learning model architecture, it is difficult to summarize the relevant laws. The selection of a network structure still mainly relies on human experience. Therefore, there is an urgent need to develop intelligent methods for automatically selecting neural network structures.

**Figure 2:**
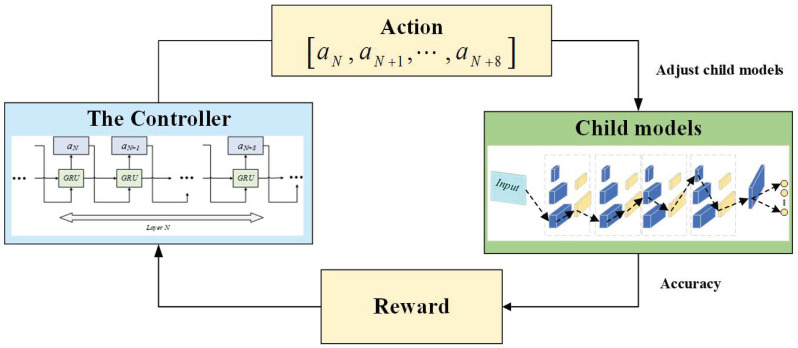
Reinforcement learning model.

In this paper, a reinforcement learning model for optimal network structure selection is presented. The reinforcement learning model, illustrated in Figure 2, can be seen as a quadruplet comprising a controller, actions, child models, and rewards. First, the controller generates a series of actions to construct the child model network structure. Then, the child model is trained using the corresponding dataset, and its classification accuracy is obtained. This accuracy is fed back to the controller as a reward. Due to the non-differentiable nature of the reward function, the controller parameters are updated using the policy gradient of reinforcement learning methods. These steps are iteratively performed to select the optimal child model. The specific structure and function of each module are explained as follows.

- Controller. The controller consists of Gated Recurrent Unit (GRU) cells, which we use to generate actions that represent the structure of the child model. The calculation process can be developed as follows:

(18) $$\begin{array}{c} z_{t}=sig\left(W_{z}\cdot \left[h_{t-1},x_{t}\right]+b_{z}\right) \end{array}$$

(19) $$\begin{array}{c} r_{t}=sig\left(W_{r}\cdot \left[h_{t-1},x_{t}\right]+b_{r}\right) \end{array}$$

(20) $$\begin{array}{c} {\tilde{h}}_{t}=tanh\left(W\cdot \left[{r_{t}*h}_{t-1},x_{t}\right]+b_{h}\right) \end{array}$$

(21) $$\begin{array}{c} h_{t}=\left(1-z_{t}\right)*h_{t-1}+z_{t}*{\tilde{h}}_{t} \end{array}$$

In which, $x_{t}$ is the input state, $h_{t}$ is the output state and $c_{t}$ represents cell state at time t. $W_{z}$ is the connection matrix for input $x_{t}$ and update gate, $W_{r}$ is the connection matrix for input $x_{t}$ and rest gate, W is the weights connected with the cell state $c_{t}$. $b_{z}$, $b_{r}$, and $b_{h}$ are the bias vectors.

**Figure 3:**
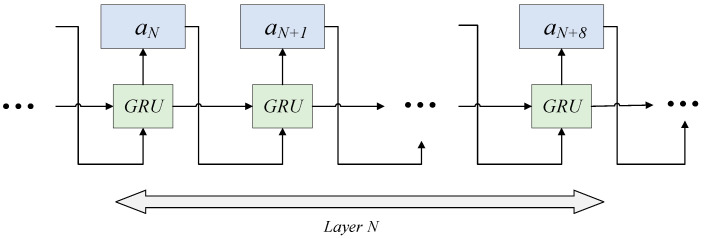
The Structure of Controller.

In this paper, the controller’s structure consists of a one-hidden-layer GRU connected to a softmax classifier. The fundamental architecture of the controller is illustrated in Figure 3. The input to the controller is the child model network structure from the previous time step, and the output consists of predicted actions used to construct the child model.

In which *θ* denotes controller parameters, the objective function $J\left(\theta \right)$ can be denoted as follows:

(22) $$\begin{array}{c} J\left(\theta \right)=-\frac{1}{8t}\sum_{t=1}^{8t}\left(\hat{P}logP\right)+\lambda \sum \theta^{2} \end{array}$$

$J\left(\theta \right)$ is the sum of cross entropy loss and regularization loss. λ represents the weight decay parameter. The exploration rate μ is 80%. t stands for the number of generated child models of the current dataset, which is set to be 20.

Currently, Adam, RMSProp, AdaDelta, and stochastic gradient descent (SGD) are commonly used optimization algorithms. However, there are currently no established guidelines for determining which optimization algorithm yields the best results. This means that the selection of the optimization algorithm is essentially based on experiments. To overcome the effects of different optimization algorithms on the selection of network structures, the controller is selected for each of the four different optimization algorithms when carrying out the selection of the four base model structures in this paper.

- Action. Actions are the laws that link the controller to the child model. Actions are generated by the controller and are used to select the sub-model network structure. After each parameter update, the controller produces a list of actions $A=\left[a_{1},a_{2},\cdots ,a_{8}\right]$, in which $\left[a_{1},a_{2}\right]$ is the value of kernels and filters in the first convolution layer, $\left[a_{3},a_{4}\right]$ is the value of kernels and filters in the second convolution layer, and the rest, $\left[a_{5},a_{6}\right]$ and $\left[a_{7},a_{8}\right]$, may be deduced by analogy.
- Child model. The child model is constructed by actions generated in the previous step. To ensure efficiency and minimize computational effort, we predefined the search space for the child model as a four-layer convolutional structure, each layer consisting of kernels and filters, with the values of the kernels chosen from [1 × 1] and [3 × 3] and the values of the filters chosen from 16, 32, and 64. Each child model contains an input layer, four-layer convolutional layers, a global average pooling layer, and a fully connected layer. At iteration t, the controller produces new actions, trains the child model with the corresponding dataset, and obtains an accuracy ${acc}_{t}$.
- Reward (R). The configuration of the reward system is pivotal for optimizing the selection of the child model structure in terms of efficiency and effectiveness. To maximize cumulative rewards, an exponentially weighted average method is employed. In this method, the accuracy obtained at each iteration is assigned different weights following an exponential decay function.

The exponentially weighted average accuracy ${EWA}_{t}$ at iteration t can be calculated as follows:

(23) $$\begin{array}{c} {EWA}_{t}=\beta^{t-1}*\left(1-\beta \right)*{acc}_{1}+\beta^{t-2}*\left(1-\beta \right)*{acc}_{1}+\\ \cdots +\beta *\left(1-\beta \right)*{acc}_{t-1}+\left(1-\beta \right)*{acc}_{t} \end{array}$$

In order to balance the current accuracy and the historical accuracy, β is selected as 0.8.

After adding the bias correction:

(24) $$\begin{array}{c} {EWA}_{t}^{\prime }=\frac{{EWA}_{t}}{1-\beta^{t}} \end{array}$$

The cumulative reward $R_{t}$ set to be ${acc}_{t}$ at t iterations minus ${EWA}_{t}^{\prime }$.

(25) $$\begin{array}{c} R_{t}={acc}_{t}-{EWA}_{t}^{\prime } \end{array}$$

To select the optimal child model, which is required to maximize the cumulative reward $R_{t}$, the objective function of the controller can be written as

(26) $$\begin{array}{c} L\left(\theta \right)=E_{P\left(a_{1:8};\theta \right)}\left[R_{t}\right] \end{array}$$

$R_{t}$ is none-differentiable, the hyperparameters θ of the controller are updated using a policy gradient method.

(27) $$\begin{array}{c} \nabla_{\theta }L\left(\theta \right)=E_{P\left(a_{1:8};\theta \right)}\left[\nabla_{\theta }J\left(\theta \right)R_{t}\right] \end{array}$$

(28) $$\begin{array}{c} \nabla_{\theta }L\left(\theta \right)=\frac{1}{t}\sum_{k=1}^{t}\nabla_{\theta }J\left(\theta \right)R_{t} \end{array}$$

To reduce variance, the bias term b is introduced:

(29) $$\begin{array}{c} \frac{1}{t}\sum_{k=1}^{t}\nabla_{\theta }J\left(\theta \right)\left(R_{m}-b\right) \end{array}$$

After adding the discount factor γ, $\nabla_{\theta }L\left(\theta \right)$ can be modified as

(30) $$\begin{array}{c} \nabla_{\theta }L\left(\theta \right)=\frac{1}{t}\sum_{k=1}^{t}\nabla_{\theta }J\left(\theta \right)\left(\gamma^{t^{\prime }-t}R_{m}-b\right) \end{array}$$

in which γ is 0.99.

### 3.2. Cross-Validation Method

Traditional training methods commonly employ the complete training dataset, which can easily lead to overfitting. Currently, cross-validation methods are applied to integrated methods, which can improve data utilization and effectively prevent overfitting and underfitting [26].

**Figure 4:**
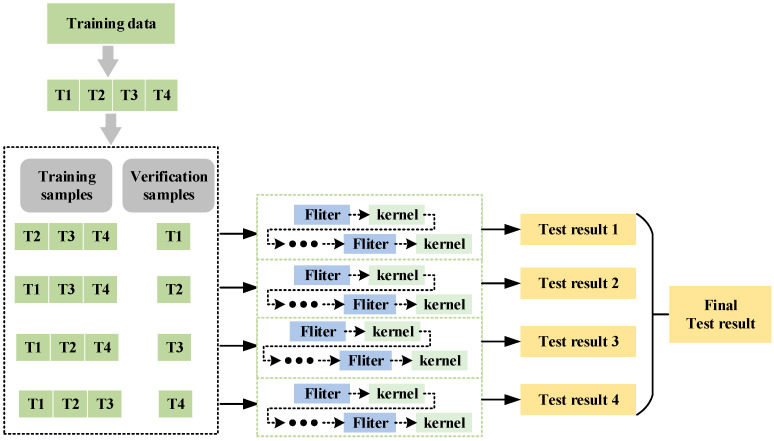
Cross-validation method.

The main concept of the cross-validation method is shown in Figure 4. Divide the original training dataset into four subsets, with three subsets serving as the training set and the other subset serving as the verification set. For each iteration, use a subset of three training sets to train the model and use the remaining subset of one verification set to validate the performance of the model. The reinforcement model is trained with four datasets, respectively, so as to obtain the optimal network structure suitable for the current dataset as the base model. Eventually, four base models with different network structures can be obtained.

The detailed process of this method is (1) the fault data samples of the same type collected in different working conditions are directly assigned with the same labels to construct the training dataset; (2) the original data are divided into training sets and a test set; (3) the reinforcement models are trained with four datasets, and the controller optimization algorithms are selected as Adam, RMSProp, AdaDelta, and SGD, respectively, to obtain the optimal network structure suitable for the current dataset; (4) the four optimal network structures serve as base models, and their output matrices from the test results are preserved as input features for the subsequent ensemble model in constructing the reinforcement learning model.

### 3.3. The Sparse Artificial Neural Network

Traditional ensemble strategies like voting methods, bagging, or boosting typically employ shallow networks for their upper layer models and attempt to enhance performance by adjusting the weights of the base models, but they have limitations. To overcome these limitations, this paper employs a stacking ensemble strategy that uses deep learning models for the upper layer. The likelihood matrix output by the base models is utilized as input features for the upper model.

**Figure 5:**
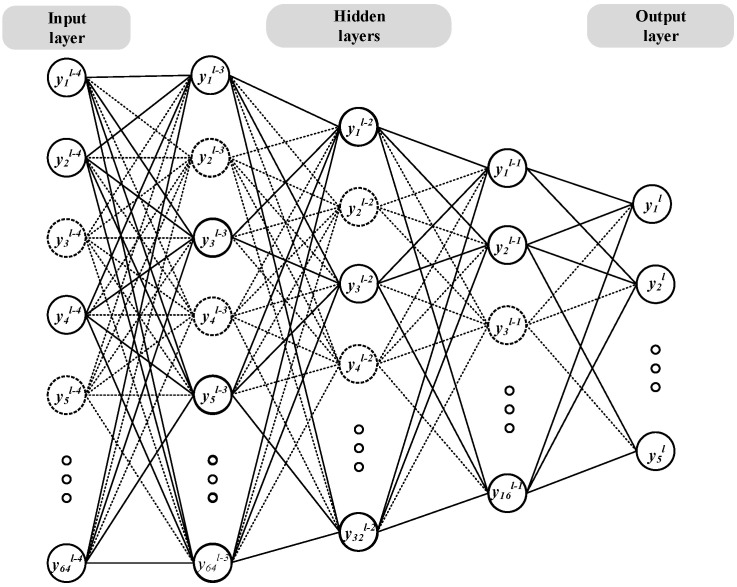
The sparse artificial neural network.

To optimize the ensemble model and prevent overfitting of the secondary learning machine, a sparse artificial neural network (SANN) is constructed as the ensemble model. The structure of the SANN is illustrated in Figure 5, comprising an input layer, three hidden layers, and an output layer.

The neural network contains four layers; the dimensionality of the input vector is 64, and the output vector is five-dimensional. The output vectors of each layer are represented as follows.

Input layer:

(31) $$\begin{array}{c} y^{l-4}={\left[y_{1}^{l-4},y_{2}^{l-4},\cdots ,y_{64}^{l-4}\right]}^{T} \end{array}$$

First hidden layer:

(32) $$\begin{array}{c} y^{l-3}={\left[y_{1}^{l-3},y_{2}^{l-3},\cdots ,y_{64}^{l-3}\right]}^{T} \end{array}$$

Second hidden layer:

(33) $$\begin{array}{c} y^{l-2}={\left[y_{1}^{l-2},y_{2}^{l-2},\cdots ,y_{32}^{l-2}\right]}^{T} \end{array}$$

Third hidden layer:

(34) $$\begin{array}{c} y^{l-1}={\left[y_{1}^{l-1},y_{2}^{l-1},\cdots ,y_{16}^{l-1}\right]}^{T} \end{array}$$

Output layer:

(35) $$\begin{array}{c} y^{l}={\left[y_{1}^{l},y_{2}^{l},\cdots ,y_{5}^{l}\right]}^{T} \end{array}$$

The weight matrix and bias vector for each layer are $w^{l-3}$, $b^{l-3}$; $w^{l-2}$, $b^{l-2}$; $w^{l-1}$, $b^{l}$ and $w^{l}$, $b^{l}$. The active functions of each layer are $f^{l-3}$, $f^{l-2}$, $f^{l-1}$ and $f^{l}$.

To address overfitting issues resulting from excessive feature learning, half of the feature detectors are randomly omitted in each batch. Consequently, during forward propagation, the activation values of specific neurons are set to 0 with a probability of P, enhancing the network’s generalization.

Thus, the expression of hidden layer one is obtained:

(36) $$\begin{array}{c} r_{j}^{l-4}\sim Bernoulli\left(p\right) \end{array}$$

(37) $$\begin{array}{c} {\tilde{y}}^{l-4}=r^{l-4}*y^{l-4} \end{array}$$

(38) $$\begin{array}{c} {net}_{i}^{l-3}=\sum_{j=1}^{64}W_{i,j}^{l-3}{\tilde{y}}_{j}^{l-4}+b_{i}^{l-3},\left(1\leq i\leq 64\right) \end{array}$$

(39) $$\begin{array}{c} y^{l-3}=f^{l-3}\left({net}^{l-3}\right)=relu\left({net}^{l-3}\right) \end{array}$$

where each element in ${net}^{l-3}$ represents the weighted sum of the input layer vector and the bias vector. Bernoulli function is used to generate the probability vector *r*, which means it randomly produces the vector of 0 or 1.

And so on, for layer l:

(40) $$\begin{array}{c} r_{j}^{l-1}\sim Bernoulli\left(p\right) \end{array}$$

(41) $$\begin{array}{c} {\tilde{y}}^{l-1}=r^{l-1}*y^{l-1} \end{array}$$

(42) $$\begin{array}{c} {net}_{i}^{l}=\sum_{j=1}^{16}W_{i,j}^{l}{\tilde{y}}_{j}^{l-1}+b_{i}^{l},\left(1\leq i\leq 5\right) \end{array}$$

(43) $$\begin{array}{c} y^{l}=f^{l}\left({net}^{l}\right)=sig\left({net}^{l}\right) \end{array}$$

During the training of the sparse network, the process involves randomly removing half of the hidden neurons while keeping the input and output neurons intact. The input features are then forwarded through the altered network, and the loss function is backpropagated through this modified network. By employing the random gradient descent method, a small set of training samples is used to update the parameters of the undeleted neurons. Subsequently, the deleted neurons are restored (during this phase, the deleted neurons remain unchanged, and the undeleted neurons are updated), and this training process is repeated until the training is complete.

### 3.4. General Procedures of Proposed Method

**Figure 6:**
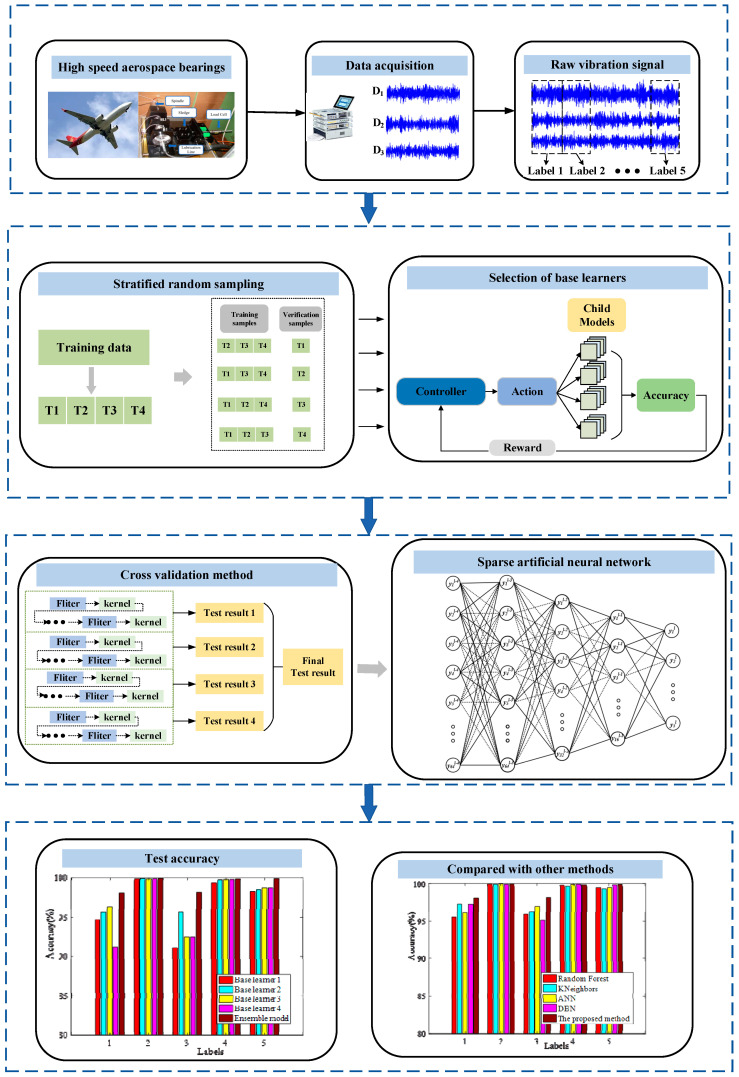
Abstract graphic of proposed method.

To address the challenges of fault diagnosis under variable working conditions, this paper presents a reinforcement ensemble method. It automatically selects base learners and combines them using a sparse artificial neural network. The schematic representation of the proposed method is depicted in Figure 6, and the detailed procedures are outlined as follows:

(1) The fault data samples of the same type collected under different working conditions are directly assigned with the same labels to construct the training dataset.
(2) The original data are divided into training sets and a test set.
(3) The reinforcement models are trained with four training datasets to obtain four optimal network structures; the controller optimization algorithms are selected: Adam, RMSProp, AdaDelta, and SGD, respectively.
(4) The sparse artificial neural network is designed as the ensemble model, and the four optimal network structures obtained in the previous step are used as the base learners.
(5) The output matrix of the base learners is used as the input feature of the ensemble model.
(6) The proposed method is verified with a high-speed aerospace bearings dataset under variable work conditions.

## 4. Experimental Verification

### 4.1. Introduction of Experimental Dataset

**Figure 7:**
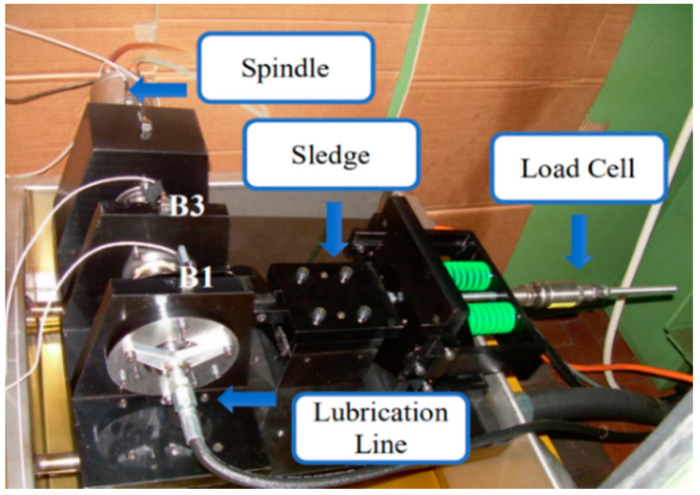
General view of the test rig.

This paper was validated through data collected from experiments conducted on a test rig established at the DIRG Lab within the Department of Mechanical and Aerospace Engineering. The test rig is purposefully designed to evaluate high-speed aerospace bearings, capturing acceleration data across varying speeds, radial loads, and damage levels. The test stand, as depicted in Figure 7, consists of a high-speed spindle with a rotating drive shaft. The spindle’s main body is securely fastened to a robust stand placed on a substantial steel base plate. The spindle’s speed is regulated via the inverter’s control panel. On the same base plate, two brackets hold two identical roller-bearing outer rings (positions B1 and B3). The inner rings of these bearings are connected to a short hollow shaft that is custom-designed for operation at speeds of up to 35,000 rpm. During the experiment, the precision sledge connected to the outer ring of the bearing is rotated and pulled by the nut, compressing two parallel springs to generate the required load [27].

The test data were gathered by measuring the acceleration of the bearing support and the electric spindle. For this purpose, a triaxial IEPE-type accelerometer was employed. This accelerometer is capable of recording frequencies within the range of 1 to 12,000 Hz (with an amplitude tolerance of ±5% and phase tolerance of ±10°). It features a nominal frequency of 55 kHz and a nominal sensitivity of 1 mV/ms^−2^.

Radial acceleration data were collected from bearings under various conditions, including different levels of damage, operating speeds, and loads. The conditions used were as follows: Nominal speed: 100 Hz, and nominal load: 0 N. Nominal speed: 200 Hz, and nominal load: 1000 N. Nominal speed: 300 Hz, and nominal load: 1800 N. Under these different operating conditions, the radial accelerations of the bearings were measured. Training and test samples were collected at a sampling rate of 51,200 Hz.

The dataset contained three different fault domains, each containing 4500 samples. Therefore, the original data consisted of 13,500 samples. To ensure the independence of the test samples. The 13,500 samples were divided into training samples and test samples, of which 9000 samples were training data and 4500 samples were test data. We divided the training data into four subsets, each containing 2250 samples, with three subsets serving as the training set and the other as the validation set. For each iteration, we used a subset of three training sets to train the model and the remaining subset of one validation set to validate the performance of the model.

**Figure 8:**
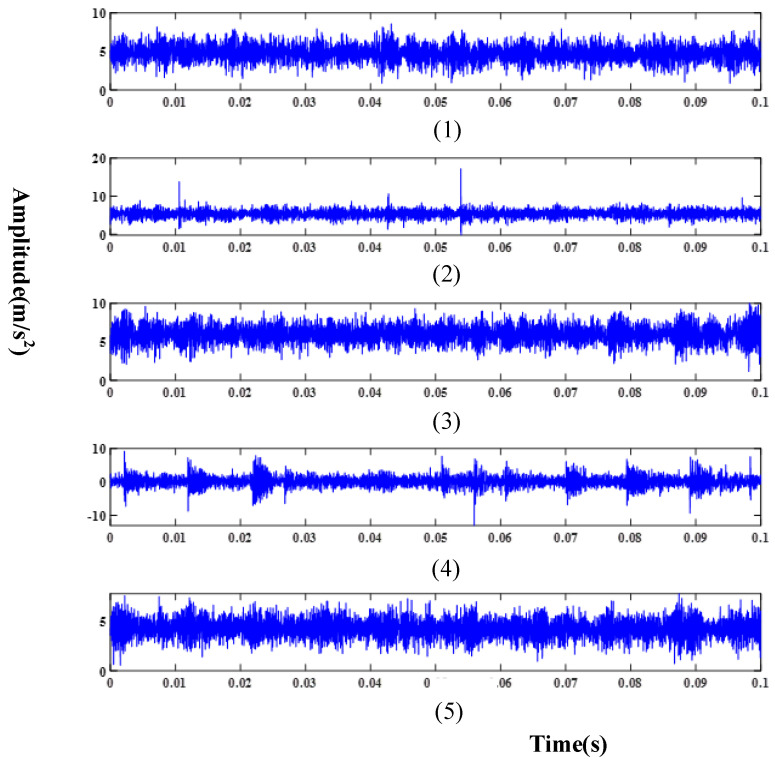
Raw signal in $D_{1}$: (**1**) Normal condition; (**2**) Serious inner ring fault; (**3**) Slight inner ring fault; (**4**) Serious roller fault; (**5**) Slight roller fault.

**Figure 9:**
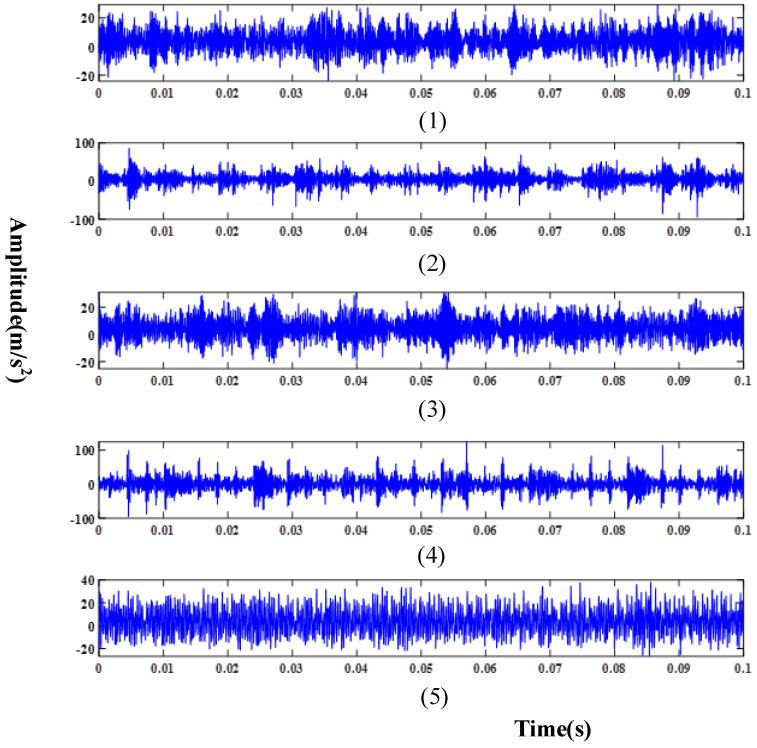
Raw signal in $D_{2}$: (**1**) Normal condition; (**2**) Serious inner ring fault; (**3**) Slight inner ring fault; (**4**) Serious roller fault; (**5**) Slight roller fault.

**Figure 10:**
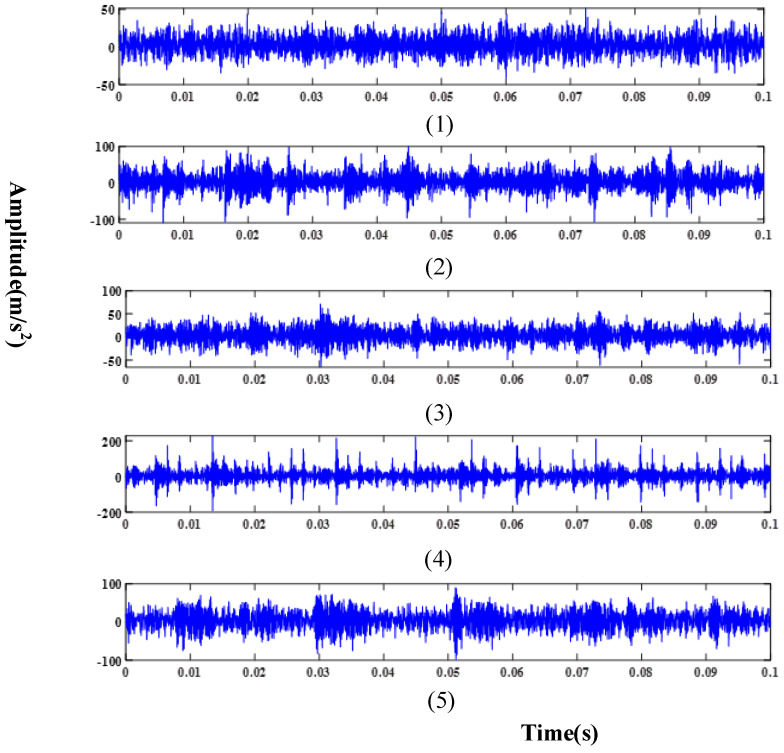
Raw signal in $D_{3}$: (**1**) Normal condition; (**2**) Serious inner ring fault; (**3**) Slight inner ring fault; (**4**) Serious roller fault; (**5**) Slight roller fault.

Meanwhile, in order to ensure the fairness of the experiment, the test set consisted of 2250 randomly selected test samples from 4500 test samples, which were used to verify the performance of the proposed method and other comparative methods. Table 1 provides details about three distinct fault domains, while Table 2 describes the various fault types within each domain. Raw signals collected under different working conditions using the accelerometer are illustrated in Figure 8, Figure 9 and Figure 10.

### 4.2. The Effectiveness of Proposed Learning

To verify the REM method proposed in this paper, four datasets were trained with the reinforcement model for 20 epochs to obtain the four base models. The base models were adopted as base learners of the ensemble model. The structures of four base learners are shown in Table 3.

**Figure 11:**
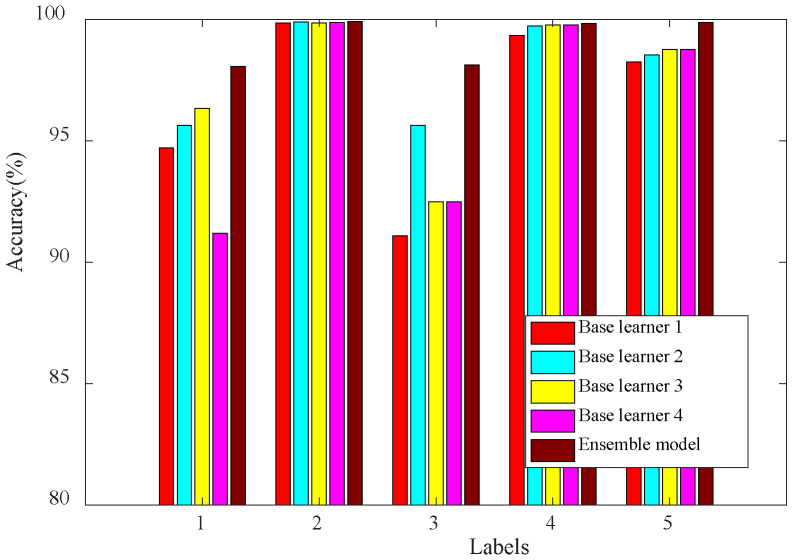
Test result of proposed method.

Using the above four convolutional models as the base learners, followed by a sparse artificial neural network (SANN) as the secondary trainer, the hidden layer structure of SANN was 64-32-16, and the activation function was the relu function. The reinforcement ensemble model was constructed and executed ten times under consistent conditions, resulting in the average accuracy presented in Table 4. The visual representation of the test results can be seen in Figure 11.

The test results illustrate that the highest accuracy among these base classifiers is of base learner two, which can reach 97.90%. Base learner one has a slightly lower accuracy than base learner two and base learner three, and the worst is base learner four, which has a test accuracy of 94.71%. Among them, the four base classifiers have better recognition of fault type two and fault type four, and the average accuracy can reach more than 99%. The next is the fifth fault type, except for base learner four, whose average accuracy is 94.71%, and the other three base learners can reach more than 98%. In contrast, the recognition accuracy of the first and third fault types is poor in general.

After ensembling the base model with SANN, the overall accuracy has improved significantly, and the average accuracy can achieve over 99%. The accuracy of the first and third types of faults can reach more than 98%, and the accuracy of the remaining three types of faults is close to 100%.

In summary, the reinforcement ensemble learning model efficiently selects the base models, and the sparse artificial neural network (SANN) as a secondary learning machine notably enhances the accuracy in recognizing various types of faults.

**Figure 12:**
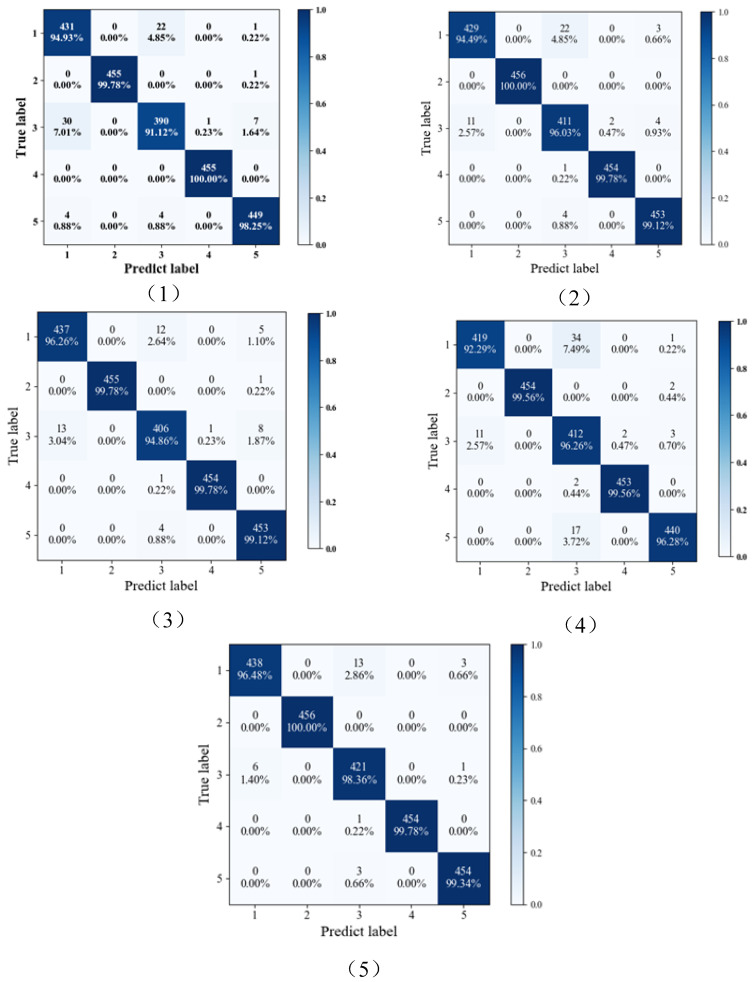
Confusion matrix of proposed method. (**1**) Base learner one, (**2**) Base learner two, (**3**) Base learner three, (**4**) Base learner four, and (**5**) Ensemble model.

The confusion matrix analysis reveals interesting patterns in the performance of the base learners. Base learner one tends to struggle with the third fault type, often misclassifying it as the first fault type. Similarly, the first fault type frequently gets misclassified as the third fault type. In contrast, the second and fourth fault types exhibit high accuracy in base learner one. Base learner two follows a similar pattern, with the first fault type having low accuracy and frequently being misclassified as the third fault type. The second, fourth, and fifth fault types achieve accuracy rates exceeding 99%. Base learner three exhibits lower accuracy for the third fault type and often mistakes it for the first fault type. The first fault type, again, is prone to being misclassified as the third fault type. Base learner four shares characteristics with the other learners, with the first fault type showing lower accuracy and often getting misclassified as the third fault type, along with the third fault type itself. The fifth fault type is also commonly misclassified as the third fault type. These insights highlight the strengths and weaknesses of each base learner when dealing with specific fault types. The ensemble model aims to leverage these differences to improve overall fault diagnosis performance. (See Figure 12).

After being ensembled by SANN, it can be found that the accuracy of both the first and third fault types improves significantly and reaches above 98%. The accuracy of the remaining three types of faults is also above 99%. The proposed method has successfully improved diagnostic accuracy and can effectively identify variable types of faults under different working conditions.

Various ensemble methods, such as the Random Forest Classifier (RF), K-Nearest Neighbor Classifier (KNN), Fully Connected Artificial Neural Network (ANN), and Deep Belief Network (DBN), were applied to combine the predictions from the four base learners selected through a reinforcement learning model. These ensemble methods had specific configurations and were trained for 100 epochs to assess their performance. Notably, the Random Forest Classifier utilized 200 decision trees, while the K-Nearest Neighbor Classifier considered six neighboring points. The Fully Connected Artificial Neural Network had a hidden layer structure of 64-32-16, and the Deep Belief Network was structured with two hidden layers and hidden units of 200, 100, and 50. The outcomes of these experiments were recorded and are summarized in Table 5.

Compared to the test results in Table 4, all five ensemble methods can improve the test accuracy of the base learners, but the proposed method is the most outstanding. The second is KN, ANN, and DBN, and the RF is slightly worse. Since the diagnostic accuracy of the base learners selected by the reinforcement learning method is relatively high, the total accuracy of the proposed method is not very prominent. However, the performance of the proposed method is significantly better than that of other integration methods in the first and third fault types, which are the most difficult to identify.

**Figure 13:**
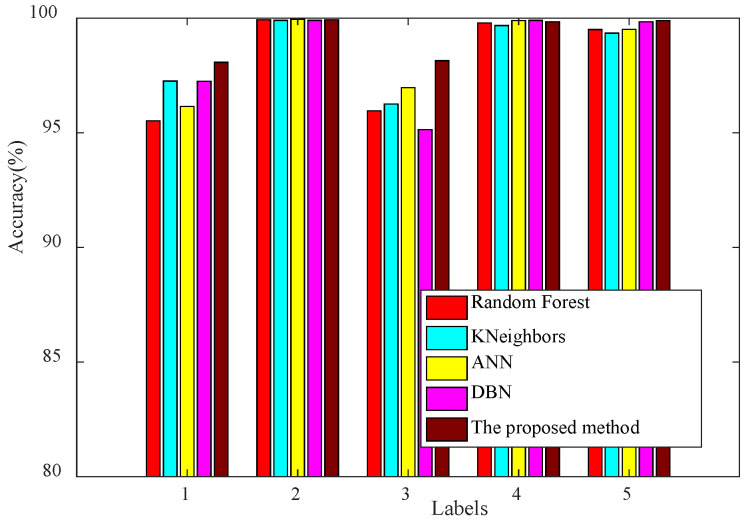
Test result of different ensemble methods.

The test results are represented visually in Figure 13. The proposed method demonstrates notable superiority, particularly in the identification of the first and third fault types, which are challenging to distinguish. In contrast, when dealing with the second, fourth, and fifth fault types, the ensemble methods’ performance exhibits marginal differences. This can be attributed to the strong individual performance of the base learners in these cases, as they consistently deliver commendable recognition results. On the whole, the method introduced in this study surpasses other integrated techniques.

**Figure 14:**
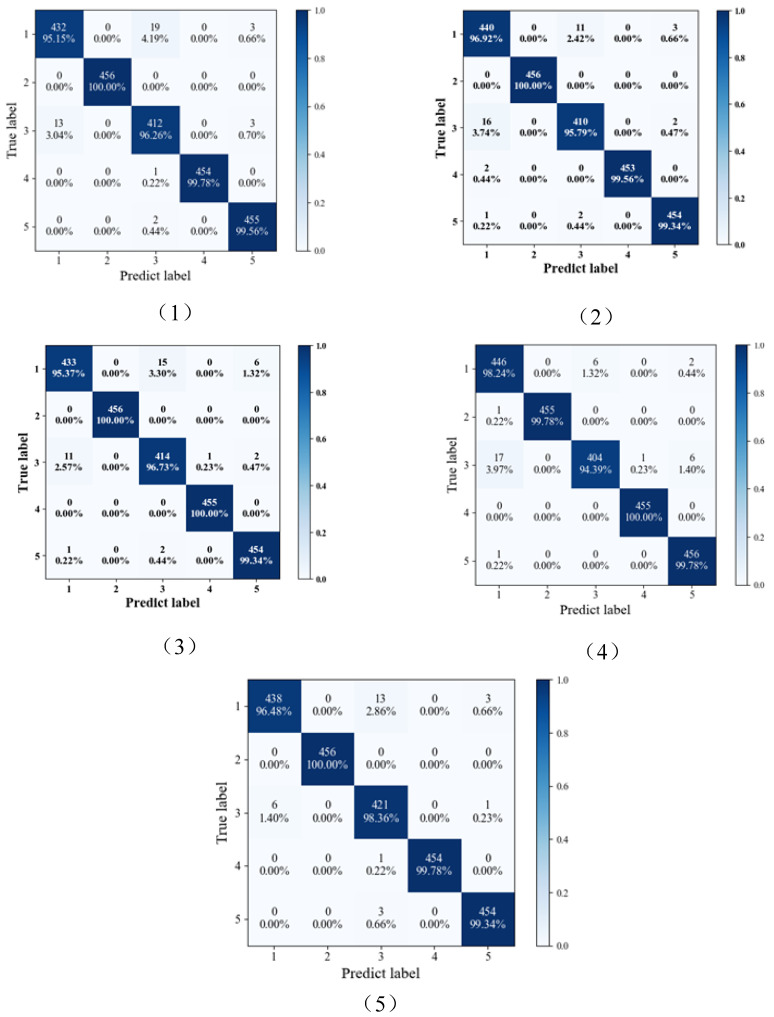
Confusion matrix of different ensemble methods (**1**) RF (**2**) KN (**3**) ANN (**4**) DBN (**5**) The proposed method.

We randomly selected a group of test results to show the confusion matrix. The confusion matrix of the test results for different ensemble methods is shown in Figure 14. It can be seen that in RF, the first type of fault has the lowest accuracy rate of 95.15% and is most likely to be misclassified as the third category of fault. This is followed by the third type of fault, which is easily misclassified as the first type of fault, and the second, fourth, and fifth types of faults have the highest accuracy, which can reach more than 99%. For KN, the third fault type has the lowest accuracy and is also prone to be misclassified as the first category of faults, followed by the first category of faults, which is prone to be misclassified as the third category of faults, and the second, fourth, and fifth category of faults can reach an accuracy of more than 99%. Similarly, in ANN, the first category of faults has the lowest accuracy rate and is most likely to be misclassified as the third category of faults. This is followed by the third fault type, which is easily misclassified as the first type of fault. Finally, in DBN, the third fault type has the lowest accuracy and is likewise easily misclassified as the first fault type, followed by the first type of fault, which is easily misclassified as the third type of fault. For the proposed method, similarly, the accuracy of the first- and third-category faults is relatively low, but the diagnostic accuracy is better than the other integration methods overall.

### 4.3. Compared with Other Intelligent Diagnosis Methods

The proposed method is evaluated in comparison with several established machine learning and deep learning methods for fault identification. Specifically, the BP network architecture consists of multiple hidden layers with the specifications of 256-128-64-32 and employs the Rectified Linear Unit (ReLU) activation function. The optimization method employed is AdaDelta. The Support Vector Machine (SVM) utilizes a regularization coefficient of 5.0 and applies the radial basis kernel function (Gaussian kernel function). For Convolutional Neural Networks (CNNs), four convolutional layers are used with corresponding kernel and filter sizes of [1,16] [3,32] and [1,16] [3,32], respectively. These convolutional layers are followed by a global average pooling layer, a fully connected layer, and a softmax classifier. LSTM (Long Short-Term Memory) is utilized with a single layer containing 128 hidden units.

Therefore, 6750 samples are randomly sampled from the original training data while keeping the test dataset unaltered. Each method underwent training 100 times, resulting in multiple sets of test results. The various methods were executed ten times under identical conditions, and the average test accuracy was calculated and is presented in Table 6.

The proposed method consistently outperforms the other approaches in terms of the average accuracy. Detecting the same fault type under varying working conditions proves challenging when employing a straightforward, intelligent diagnostic model. Among them, the diagnosis effect of the CNN and BP neural networks is better than that of SVM and LSTM, and the overall recognition accuracy can reach 87%. However, for the first, third, and fifth types of faults, the recognition accuracy is low, especially for the third type of faults, and the recognition accuracy of these two methods does not reach 70%. The diagnosis results of SVM and LSTM are poor at 75.82% and 77.93%, respectively. These two methods have poor recognition results for the third and fifth types of faults. For the first type of fault, the diagnosis accuracy of SVM can reach 94.05%, while LSTM is only 70.16%. However, these four intelligent methods can accurately identify the second and fourth types of faults. The proposed method effectively and accurately identifies various types of faults, achieving an overall recognition accuracy exceeding 99%, thereby demonstrating its efficacy.

**Figure 15:**
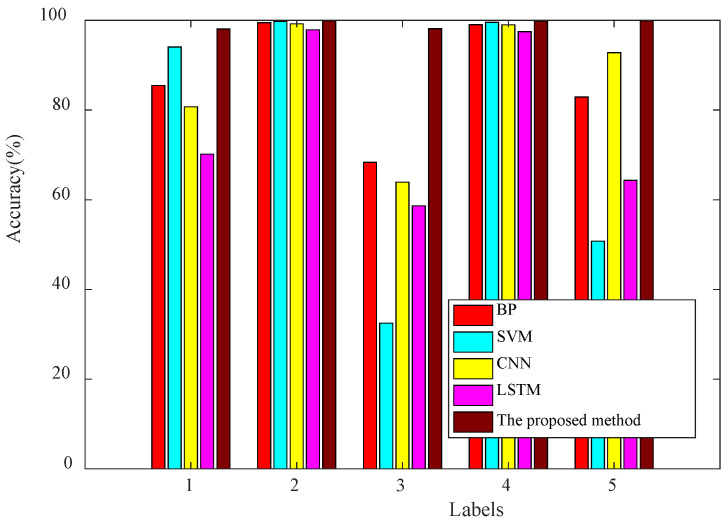
Test result of different diagnosis methods.

Figure 15 visually demonstrates the diagnostic accuracy of these methods, with the proposed approach consistently exhibiting high accuracy in identifying various fault types. The other four intelligent diagnosis methods can only accurately identify the second and fourth types of faults. For the BP network, the identification accuracy of the third fault type is the worst, followed by the fifth type of fault. The identification accuracy of the first fault type is slightly poor, but it can also reach 85%. The accuracy of the third and fifth types of fault recognition of SVM is very low. CNN also has the lowest accuracy rate for the third type of fault, followed by the first type of fault. LSTM has poor accuracy for three types of faults that are difficult to identify.

### 4.4. Parameters Sensitivity Analysis

In the proposed approach, the hyperparameter μ controls the exploration rate during child model generation. And β represents the forgetting coefficient to balance the current reward and the historical reward. The values of μ and β directly affect the diagnostic result of the proposed method. Therefore, this section conducted sensitivity analysis experiments to check the rationality of the two hyperparameters. The range of μ is {50%, 60%, 70%, 80%, 90%}, and the range of β is {0.5, 0.6, 0.7, 0.8, 0.9}.

**Figure 16:**
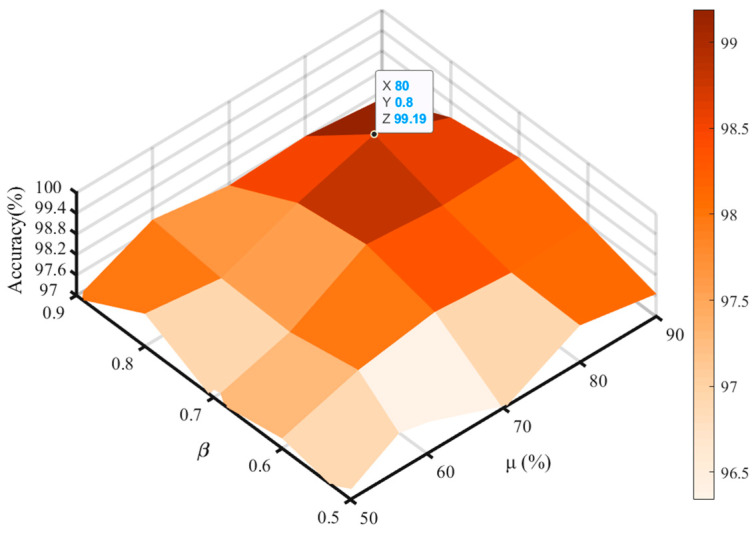
Sensitivity analysis of μ and β.

We conducted experiments on cross-domain fault diagnosis cases. Figure 16 illustrates the sensitivity analysis regarding the diagnostic outcomes and two hyperparameters. The experimental findings highlight the significant influence of hyperparameters μ and β on the diagnostic outcome. Notably, for μ = 80% and β = 0.8, the diagnostic accuracy reached its peak at 99.19%. These results underscore the validity of the selected hyperparameter values.

## 5. Conclusions

A reinforcement ensemble method was proposed for fault identification under different working conditions. Firstly, a reinforcement model was constructed to select optimal base learners. Secondly, stratified random sampling was used to extract four datasets from raw training data. The reinforcement model was trained by these four datasets, respectively, and four optimal base learners were obtained. Finally, a sparse ANN was designed as the ensemble model, and the reinforcement learning model that could successfully identify the fault type under variable work conditions was constructed.

The proposed method has been rigorously tested using a high-speed aerospace-bearing dataset. The results from these tests demonstrate the method’s ability to effectively identify identical fault modes under varying working conditions. This achievement marks a departure from the previous approaches that relied on specific data distributions, instead leveraging deep learning models to identify consistent fault characteristics. Going forward, the authors plan to refine the reward function further to achieve global optimization of the model.

## Figures and Tables

**Table 1:** Details about different fault domain.

Fault Domain	Nominal Speed (Hz)	Nominal Load (N)	Training Samples	Test Samples
$D_{1}$	100	0	2250	750
$D_{2}$	200	1000	2250	750
$D_{3}$	300	1800	2250	750

**Table 2:** Descriptions of each fault conditions.

Fault Types	Fault Dimension (μm)	Training Samples	Test Samples	Labels
Normal condition	0	450	150	1
Inner ring fault	450	450	150	2
Inner ring fault	150	450	150	3
Roller fault	450	450	150	4
Roller fault	150	450	150	5

**Table 3:** Base learners earned by meta learning method.

Structure	Base Learners			
	Base Learner 1	Base Learner 2	Base Learner 3	Base Learner 4
Convolution layer (filters, kernels)	(64, 3)	(64, 3)	(16, 3)	(32, 3)
Convolution layer (filters, kernels)	(16, 1)	(64, 3)	(64, 3)	(32, 3)
Convolution layer (filters, kernels)	(64, 3)	(32, 3)	(64, 3)	(64, 3)
Convolution layer (filters, kernels)	(32, 3)	(32, 3)	(16, 3)	(16, 1)
Pooling layer	2 × 2	2 × 2	2 × 2	2 × 2
Output layer	5	5	5	5
Optimization algorithms	Adam	RMSProp	AdaDelta	SGD

**Table 4:** Testing result of proposed method.

Models	Accuracy					
	Labels					Total Accuracy
	1	2	3	4	5	
Base learner 1	94.71%	99.86%	91.09%	99.34%	98.25%	96.71%
Base learner 2	95.63%	99.91%	95.65%	99.74%	98.55%	97.29%
Base learner 3	96.33%	99.87%	92.49%	99.78%	98.76%	97.51%
Base learner 4	91.19%	99.88%	93.77%	99.52%	94.71%	95.84%
Ensemble model	98.07%	99.93%	98.14%	99.84%	99.89%	99.19%

**Table 5:** Testing result of different ensemble methods.

Models	Accuracy					
	Labels					Total Accuracy
	1	2	3	4	5	
RF	95.52%	99.92%	95.95%	99.78%	99.50%	98.13%
KN	97.25%	99.90%	96.25%	99.67%	99.34%	98.48%
ANN	96.15%	99.95%	96.96%	99.89%	99.51%	98.49%
DBN	97.24%	99.90%	95.13%	99.90%	99.84%	98.41%
The proposed method	98.07%	99.93%	98.14%	99.84%	99.89%	99.19%

**Table 6:** Testing result of different diagnosis methods.

Models	Accuracy					
	Labels					Total Accuracy
	1	2	3	4	5	
BP	85.47%	99.48%	68.36%	99.04%	82.93%	87.28%
SVM	94.05%	99.78%	32.48%	99.56%	50.77%	75.82%
CNN	80.73%	99.23%	63.91%	99.01%	92.78%	87.42%
LSTM	70.16%	97.90%	58.65%	97.47%	64.33%	77.93%
The proposed method	98.07%	99.93%	98.14%	99.84%	99.89%	99.19%

## Data Availability

The original data presented in the study are openly available at DOI 10.1016/j.ymssp.2018.10.010.
